# Eating and Lifestyle Habits in Youth With Down Syndrome Attending a Care Program: An Exploratory Lesson for Future Improvements

**DOI:** 10.3389/fnut.2021.641112

**Published:** 2021-09-08

**Authors:** Giulia Roccatello, Guido Cocchi, Rosa Tullia Dimastromatteo, Alessandra Cavallo, Giovanni Battista Biserni, Mariella Selicati, Maria Luisa Forchielli

**Affiliations:** ^1^Consultant Registered Dietitian, Ass Diet I., Bologna, Italy; ^2^Pediatrics, University of Bologna, Bologna, Italy; ^3^Health Science and Technologies Interdepartmental Center for Industrial Research (CIRI-SDV), University of Bologna, Bologna, Italy

**Keywords:** nutrition, multidisciplinary approach, prevention, obesity, lifestyle, down syndrome, children

## Abstract

**Introduction:** Children with Down Syndrome (DS) have nutritional problems with unknown implications besides increased potential for obesity. Their food habits are unknown. We aim to delineate eating and lifestyle habits of DS children attending a multispecialist program to identify the challenges they face and the potential improvements.

**Patients and Methods:** We interacted with 34 DS children (22 males, 12 females, 2–16 years old) and their families. Food habits, medical conditions and treatments, degrees of development and physical activity, anthropometric and laboratory data were recorded over 6 months and analyzed. A 3-day food diary and a 24-h recall food frequency questionnaire were administered.

**Results:** Twenty-nine (85%) children completed meals, only 11 (32%) received alternative food such as milk. Weaning regularly started in 25 (73%) children. Preschool children introduced adequate calories and nutrients. School children and adolescents did not reach recommendations. All age groups, as the general pediatric population, excessively ate protein and saturated fat, and preferred bread, pasta, fruit juices, meat and cold cuts. Peculiarly, pulses and fish were adequately assumed by preschool and school children, respectively. Five children (15%) were overweight/obese.

**Conclusions:** Dietary excesses commonly found in the general pediatric population are also present in this DS group, proving a narrowing gap between the two. DS group performed better nutritionally in the early years and overweight/obesity occurrence seems contained. DS children may benefit from a practical yet professional care-program in which nutrition education may improve their growth, development and transition into adulthood.

## Introduction

Down Syndrome (DS) is a common gene alteration syndrome, mostly consisting in an anomaly in the 21st chromosome. In Europe, DS children are 8% of registered cases of congenic anomalies. Worldwide, prevalence is 23 out of 10,000 births, with a tendency to increase (1). DS people increased their life expectancy, going from 25 years in 1983 to ~60 in 2010, despite their susceptibility to congenital diseases and genetic predisposition to multiple medical conditions such as thyroid and dental problems, leukemia, cardiac or gastrointestinal defects, respiratory infections, pulmonary hypertension, immune dysregulations, ocular and auditory disorders, dermatological diseases, and epilepsy (2, 3). Numerous studies have attempted to find therapies, but very few have investigated the food habits of this population (4–6). Macro- and micro-nutrients, diet composition, habits and lifestyles may be fundamental to sustain good health, especially if approached in the 1st years of life. Proper nutrition from the periconceptual stage (first 1,000 days) may effectively prevent or delay the onset of numerous diseases in childhood and especially in adulthood (2). Dietary antioxidants are essential for DS children, as the condition exposes them to multiple deficiencies and to fast aging. Eating is not always easy, due to swallowing and chewing difficulties (6, 7). In some infants, cardiopathy may impair food tolerance; in others, unfavorable upper airway anatomy may increase the frequency of aspiration. These difficulties tend to worsen with time, although adults often “outgrow” them (8).

To fill the gap between diet and health in DS children and to explore potential key factors for improvement, we decided to scour their eating habits, their qualitative and quantitative dietary patterns in relation to their medical conditions and lifestyles, and to compare their food intakes with recommendations for the general pediatric population. Comparisons with what available in the literature for the DS population will also be considered as well as the effects of the participation to this small care program.

## Methods and Patients

Thirty-four children, 22 males and 12 females, median age 7 years (range 2–16 years), 31 of Italian nationality and 3 from other countries (2 from Morocco, and 1 from the Philippines), were consecutively recruited over a period of 6 months and followed by a dietician while they were participating in their therapeutic sessions at the “Insieme Club” (a non-profit organization for children with disabilities affiliated to the Antoniano, Bologna, Italy). The Club offers a 360° support consisting of medical care, speech therapy, nutrition counseling, physiotherapy, art and musical education. The children are involved in all the activities, which are tailored to their needs. The sessions, organized in the afternoons, are group-therapy meetings, while others are one-to-one (e.g., during assessment of food provision or speech therapy). The children's parents signed an informed consent for inclusion in the study. The study was conducted in accordance with the Declaration of Helsinki and the protocol was approved by the Ethics Committee of the Antoniano Insieme Club (11/10/12). The group reflected the number of children attending the club during the study's timeframe. All the children completed the study.

After enrollment, families received a questionnaire and had face-to-face interviews with the dietician. She interacted with all the families at least three times: before handing the questionnaire to explain it including portion-size and food frequencies, after when a concomitant food recall of the previous 24 h was completed, and later when data were presented and discussed. In case of need, further assistance was offered through phone calls and extra meetings. The questionnaire was the same for all children and contained 29 items organized in five sections, as follows:

(1) Descriptive data about the child: sex; date of birth; place of birth; weight, height, and body mass index (BMI) as recorded during the interview and converted in percentiles using two different DS-specific charts and one for the general pediatric population (9–11).
(2) Quantity, quality, and distribution of food, physical activity, and relationship with food, as recorded over 3 days (one of which during the weekend). This information was crosschecked with a food recall of the previous 24 h at the time of the interview, to make sure that daily and weekly food intake frequencies were accurately recorded. Data were converted into portions and analyzed using the Scotti-Bassani Food Atlas (12) to obtain average caloric intakes, distribution of calories in macronutrients, protein and fiber intakes along with cholesterol, sodium, calcium, zinc, and folic acid (nutrients of which we could have corresponding laboratory levels).
(3) Number of medical and speech-therapist follow-up visits requiring specific nutritional interventions in case of difficulties with specific food texture consumptions. Children were classified based on one or more texture difficulties (liquid, puree, mashed, solid, hard, fibrous).
(4) Medical and surgical comorbidities (e.g., constipation, obesity, gastroesophageal reflux, congenital problems).
(5) Laboratory tests: lipid profile consisting of total- (TC), high-density (HDL), and low-density lipoprotein (LDL) cholesterol (mg/dL) as well as triglycerides (TG) (mg/dL), hemoglobin (g/dL), white blood cells (/microL), total proteins (g/dL), creatinine (mg/dL), iron (mcg/dL), blood glucose (mg/dL), uric acid (mg/dL), zinc (mcg/mL), magnesium (mg/dL), calcium (mg/dL), potassium (mEq/l), sodium (mEq/l), chloride (mEq/l), vitamin B12 (ng/ml), and folic acid (ng/mL).

Data were extrapolated from the questionnaires, validated, and merged with information from the 24-h dietary recall, and then profiled according to some targeted variables which were then compared with benchmark values of the general pediatric populations or people with DS. Direct contacts with the families allowed to verify data in case of discrepancies.

### Statistical Analysis

Descriptive statistics were expressed as means, percentages, and standard deviations (SD) or ranges. Children were divided into 3 age groups: 2–6 (13 patients), 7–12 (16 patients), and 13–16 (5 patients) years. Male and female adolescents were not categorized in two separate groups because pubertal peak growth is generally delayed. Measured nutrient data were compared with the Italian recommendations (LARN, 13); expected intakes for each nutrient were computed in an individualized manner based on age, gender, and anthropometric parameters. Desired daily energy expenditure was computed using the Schofield equation (SACN, 14) with the specific four factors (age, gender, weight, and height of each child) multiplied by the lowest (25 percentile) age-range physical activity factor (13). Macronutrients were extrapolated from the desired daily energy intake following an age-specific percentage distribution: 15% of calories for proteins, 55% for carbohydrates, and 30% for lipids above 3 years of age; 15% for proteins, 50% for carbohydrates, and 35% for lipids up to 2 years of age. Protein intake was estimated based on the LARN population reference intake per kilogram per day (14). The expected fiber intake was extrapolated from the expected daily caloric intake, taking into consideration the recommended LARN index of 8.4 grams per 1,000 kilocalories. Similarly, the expected cholesterol intake was estimated using the recommended parameter of 100 mg per 1,000 kilocalories. Estimated lipids were divided into saturated and mono + polyunsaturated based on a 1:2 ratio. The benchmark for sodium was the LARN tolerable upper intake level, while the population reference intake was used for zinc, calcium, and folate.

Actual and expected intakes were compared using paired *t*-tests for the equality of means. This test was chosen because each patient's actual intake is compared with recommendations that are specific to that patient based on age, gender, weight, and height if needed. Paired *t*-tests were also used to compare the anthropometric measurements with three alternative benchmarks, two related to the pediatric DS populations and one to the general pediatric populations (9–11). In all comparisons, a *p* < 0.05 was considered statistically significant.

## Results

### Anthropometric Data

Average weight fell within the 49.5th percentile ± 28.4 standard deviation of the pediatric most recent DS population growth charts (10) and 54 ± 24 using the old DS percentiles (9). Average height also exceeded the pediatric DS population's median (59th percentile ± 30 using the recent growth charts and 62 ± 27 with the old charts). Compared to the general pediatric population growth charts (11), the children's average weight was slightly below the median (39th percentile ± 34), while average height was within the first quartile (18th percentile ± 21). Eighteen (53%) DS children did not exceed the 10th height percentile for the general pediatric population, while only 5 (15%) were at or above the 50th percentile. As for BMI, the average value corresponded to the 67th percentile (± 28) for the general pediatric population, with 9 cases (26%) in the overweight/obese area, and 3 (9%) slightly underweight. Using the pediatric DS charts, conversely, BMI values resulted normal (average percentile 44th ± 30 based on reference 10 and 48th ± 25 using reference 9) with 5 overweight/obese children (15%). No underweight children were detected using the DS specific charts. All anthropometric data were statistically different (p < 0.01) from each of the three benchmarks, with the exception of height, whose difference from the two DS-specific benchmarks was statistically insignificant.

### Favorite vs. Least-Liked Food

Pasta (82%), bread and derivatives (47%), and sweets (29%) were the favorite food. The least-liked ones were vegetables (34%) or, in specific cases, selected vegetables, followed by sweets (26%) and fruit (18%).

### Water Consumption

Average water consumption was 1 liter ± 400 ml. Children up to 3 years of age were excluded as several of them still used milk as a main source of liquids.

### Dietary Supplements

Multivitamin supplements were prescribed to 4 (12%) children by the pediatrician. One of them received an additional Omega3 supplement.

### Relationship With Food

**Figure 1:**
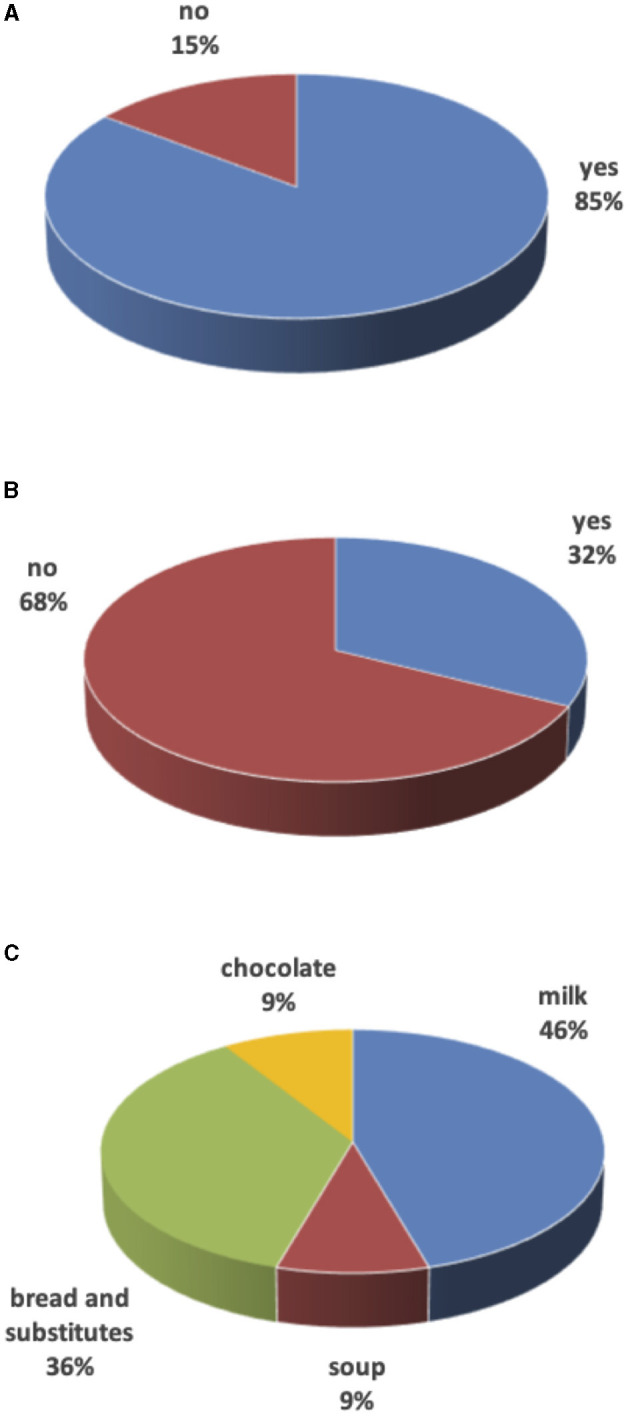
Unfinished meals. **(A)** Are meals finished? **(B)** Food integration after unfinished meals? **(C)** Food to integrate unfinished meals.

Most of the children (29, 85%) completed the portion size. Eleven (32%) ate less than half of the amount offered at meals. Alternatives were offered such as salted foods (bread and derivatives) in older children and milk in toddlers. Figure 1 summarizes overall data.

### Oral Support

Oral issues may greatly impair childhood nutrition. All children but one were followed by a dentist and 30 (88%) by a speech therapist to improve language and swallowing performances.

### Sport and Physical Activity

Most children (22 out of 29, 76%) practiced a sport outside of school. Of these, the majority devoted at least 2 h per week to physical activity, practicing more than one sport. Overall, the children were very active both at home and at school.

In both preschool and school children, average daily time on screens was less than an hour.

### Weaning and Use of the Cup

In 25 children (73%), weaning started at 7.5 ± 2 months of age and continued with a regular progressive insertion of different foods.

**Figure 2:**
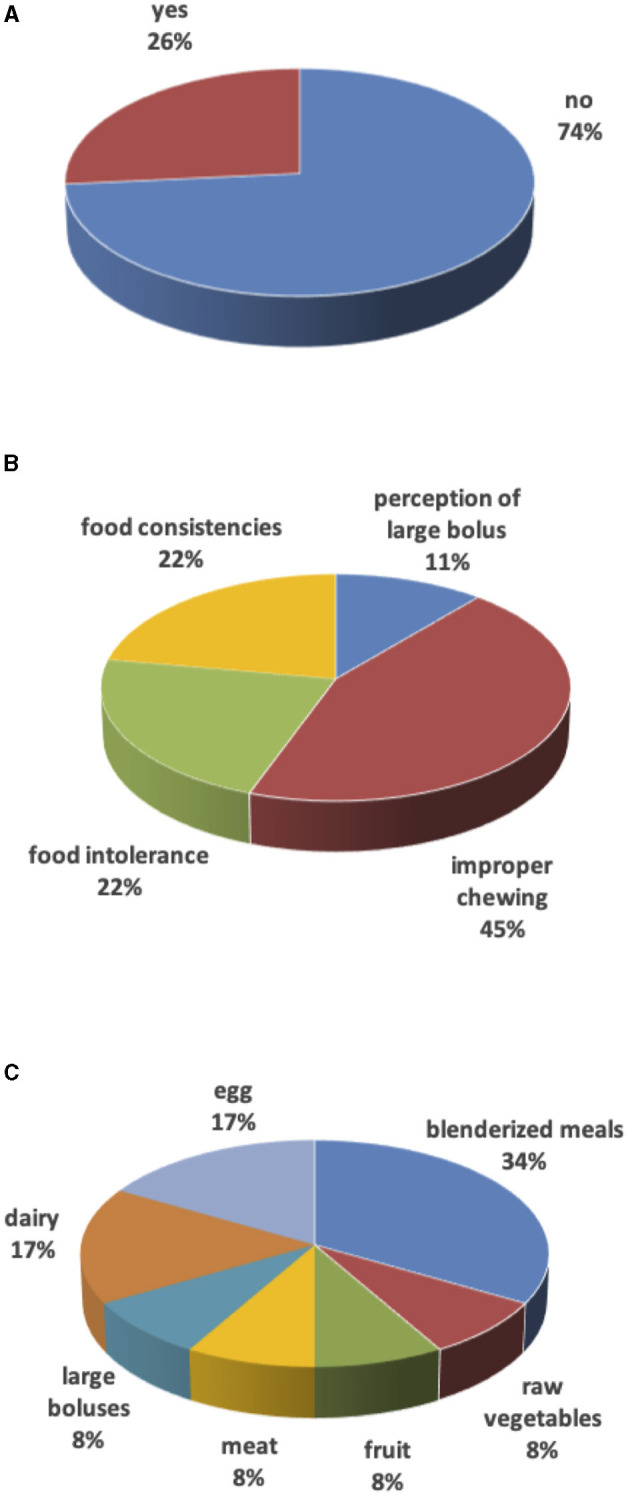
Weaning. **(A)** Weaning delay. **(B)** Reasons for weaning delay. **(C)** Food causing weaning delay.

In the remaining 9 (27%) cases, weaning was delayed due to surgery in the 1st months of life (mostly for cardiac causes) with long hospital stay, forcing families to use milk as a source of primary nourishment. These families (besides two having dairy and eggs intolerance) also reported difficulties with food textures: 4 children consumed only purees until 3–4 years of age, 1 child considered all foods to be too large in size, and 2 children had difficulties with meat, raw vegetables, or fruit. For 45% of parents, the weaning delay was due to a “lazy” chewing action (Figure 2).

Sippy cups or glasses were introduced at an average age of 23 ± 16 months; one child waited until 7 years of age. Only 7 children (21%) independently drunk from the sippy cup or glass by the recommended age limit of 24 months.

Families were also asked if they had received any nutrition counseling after weaning. The majority (53%) responded negatively, while the remaining 47% seemed to have different sources of counseling such as pediatricians and other health-care professionals, sports instructors, the Internet, and other parents. Those who joined the Club at an early age of the child were those who reached results more quickly.

### Food Consistency

Liquid and puree/mashed foods were easily ingested. As for other consistencies, 52% of parents confirmed difficulties with solid-hard consistencies such as raw vegetables or dried fruit, 48% of children had problems with dual consistencies such as pasta and beans or minestrone with pasta as well as unpeeled fruit or vegetables, and 45% with fibrous, sticky, or smelly foods such as meat, vegetables with high cellulose content, ham, and fish. The most frequent cause of complete weaning delay was lack of appetite and unwillingness to chew (50% of cases), followed by refusal of a despised taste (18%) or hard to chew (18%). In the remaining cases (14%), technical difficulties were likely to be the primary cause (orthodontic appliances, no teeth, previous surgical interventions).

### Medical Diseases and Medications

The most frequent medical problems were skin conditions such as recurrent dermatitis and eczema (35%), constipation (29%), cardiac defects (29%), diarrhea (24%), dental cavities (20%), and hypothyroidism (18%). Among drugs, the most prescribed was thyroxine, followed by polyethylene glycol.

### Laboratory Tests

Blood values, as summarized in Table 1, were found to be within normal age and gender-matched ranges, apart from zinc and TG. Zinc levels ranged from 7.8 to 17.2 μmol/L, with 10 children below the lower limit of 9.2 μmol/L and 3 above the upper limit of 17 μmol/L. TG values progressively increased during the first decade of life and significantly decreased in adolescence (*p* < 0.01).

**Table 1:** Summary of the most common laboratory values in DS children.

**Variables (normal range)**	**N patients**	**Mean**	**Standard deviation**
Hemoglobin (11–13 g/dL)	34	13	1
Blood sugar (65–110 mg/dL)	34	76	9
Ferritin (7–140 mcg/L)	34	52	35
Total Cholesterol (<200 mg/dL)	34	167	27
Triglycerides (<90 mg/dL)	34	88	44
High-density lipoprotein Cholesterol (>40 mg/dL)	34	53	14
Total protein (6.3–7.9 g/dL)	34	6.9	0.5
Sodium (137–145 mmol/L)	34	140	3
Potassium (3.6–5.2 mmol/L)	34	4.6	0.4
Chloride (102–112 mmol/L)	34	105	2
Vitamin B12 (200–900 mcg/dL)	34	534	219
Folate (3–20 mcg/L)	34	9.2	4
Magnesium (1.6–2.6 mg/dL)	34	2.2	0.1
Zinc (9.2–17 micromol/L)	34	11.8	5
Uric acid (2.5–5 mg/dL)	34	4.2	1

### Nutritional Parameters

The main results, for the entire sample and by age category, are summarized in Table 2. Overall, children ingested a surplus of protein and saturated fat, while they did not reach the recommended intakes for calcium and fiber. Adolescents introduced less calcium and folate than required. Folate was also not properly assumed by school children, while preschoolers seemed to reach the relevant recommendations. Zinc appeared to be properly introduced by all children. Caloric intake exceeded recommendations in preschool children, were in line in school children, and fell below in adolescents.

**Table 2:** Summary of nutritional intakes and comparison with desirable levels.

	**All patients (*N* = 34)**	***p*-value of paired *t*-test**	**Patients aged 1–6 (*N* = 13)**	**Patients aged 7–12 (*N* = 16)**	**Patients aged 13–16 (*N* = 5)**
	**Mean ± SD**		**Mean ± SD**	**Mean ± SD**	**Mean ± SD**
**Weight (kg)**	**29** **±** **16**		**12** **±** **2**	**24** **±** **6**	**52** **±** **7**
**Height (cm)**	**116** **±** **23**		**85** **±** **8**	**113** **±** **10**	**148** **±** **6**
**Actual protein intake (g/d)**	**62** **±** **15**	0.000	**57** **±** **6**	**63** **±** **18**	**63** **±** **13**
*Desired protein intake (g/d)*	*28 ± 15*		*12 ± 2*	*23 ± 6*	*50 ± 6*
**Actual calories intake (kcal/d)**	**1626** **±** **296**	0.903	**1348** **±** **65**	**1664** **±** **340**	**1749** **±** **158**
*Desired calories intake (kcal/d)*	*1610 ± 593*		*937 ± 210*	*1452 ± 261*	*2469 ± 185*
**Actual fiber intake (g/d)**	**12** **±** **4**	0.109	**8** **±** **4**	**11** **±** **4**	**16** **±** **3**
*Desired fiber intake (g/d)*	*14 ± 5*		*8 ± 2*	*12 ± 2*	*21 ± 2*
**Ratio of fiber intake to actual calories intake (g/1,000 kcal)**	**7** **±** **2**	0.000	**6** **±** **3**	**7** **±** **2**	**9** **±** **1**
*Desired fiber intake to actual calories intake based on recommendation (8.4 g/1,000 kcal)*	*14 ± 2*		*11 ± 1*	*14 ± 3*	*15 ± 1*
**Actual cholesterol intake (mg/d)**	**213** **±** **110**	0.065	**141** **±** **27**	**246** **±** **137**	**193** **±** **39**
*Desired cholesterol intake (mg/d)*	*130 ± 60*		*104 ± 15*	*148 ± 76*	*110 ± 15*
**Actual saturated fat intake (% of total calories)**	**20** **±** **8**	0.000	**18** **±** **4**	**22** **±** **8**	**16** **±** **8**
*Desired saturated fat intake (% of total calories)*	*10 ± 0*		*10 ± 0*	*10 ± 0*	*10 ± 0*
**Actual sodium intake (mg/d)**	**1723** **±** **923**	0.286	**1127** **±** **686**	**1490** **±** **666**	**2696** **±** **990**
*Desired upper level of sodium intake (mg/d)*	*1506 ± 389*		*1000 ± 0*	*1456 ± 251*	*2000 ± 0*
**Actual calcium intake (mg/d)**	**678** **±** **399**	0.007	**804** **±** **494**	**701** **±** **428**	**532** **±** **311**
*Desired adequate calcium intake (mg/d)*	*1069 ± 215*		*700 ± 0*	*1089 ± 93*	*1300 ± 0*
**Actual zinc intake (mg/d)**	**8** **±** **3**	0.387	**8** **±** **3**	**7** **±** **3**	**10** **±** **3**
*Desired adequate zinc intake (mg/d)*	*7 ± 3*		*3 ± 0*	*7 ± 2*	*10 ± 1*
**Actual folate intake (mcg/d)**	**196** **±** **89**	0.097	**261** **±** **163**	**156** **±** **57**	**238** **±** **42**
*Desired adequate folate intake (mcg/d)*	*249 ± 89*		*140 ± 0*	*234 ± 58*	*363 ± 25*

*SD, standard deviation*.

*The table presents actual patient data (boldfaced) and compares them with the relevant benchmarks (italic)*.

*Benchmarks were computed specifically for each patient based on age, gender, and anthropometric measurements. The table shows the relevant means and standard deviations*.

*P-values refer to paired t-tests between actual patient data and the relevant patient-specific benchmarks over the entire sample (N = 34)*.

*Means and standard deviations for the three age-based subsamples are provided for further reference. Age is measured in years*.

## Discussion

Proper nutrition, especially in the 1st years of life, is essential for a prospective healthy life. Some feeding problems in DS children have already been described (4–6). However, the longitudinal effects of nutrition and medical problems on lifestyles of DS children are unknown.

Most DS families look for help to offer a higher quality of life to their children and often they look outside of hospital settings. Therefore, we recruited a group of DS children with access to a multidisciplinary support in a familiar environment. We collected a wide range of objective data (considering the high level of knowledge these families have) through which we identified some patterns potentially useful for further investigations.

Our first consideration relies on an outcome of eating habits: anthropometric measurements. The literature repeatedly reports that DS children are predisposed to obesity (4, 15, 16). The DS prevalence of overweight and obesity has been reported to range between 23 and 70% in both males and females and to stem from altered dietary intakes, reduced physical activity, and a basal metabolic rate below that of the general pediatric population (4, 9, 15–17). Physical activity does not only produce a caloric burst; it also seems to positively impact the ability to perform daily tasks and thus improve independence in DS adults (18). In our study, being overweight/obese was not a main issue, as only 5 (15%) children could be qualified as such when the DS-specific chart was used (38% of children resulted overweight/obese on the basis of the general pediatric percentiles). This result emphasizes the need to use proper growth charts as the DS height targets are generally lower than those of the general pediatric populations. In this group, heights reached good levels despite the fact there was a slightly decreasing trend when the most recent DS growth charts were used. This may reflect the genetic and environmental factors arising from the American DS population and their evolution over 25 years (9, 10). Unfortunately, Italian DS growth charts are not available. Therefore, we selected the American old and recent DS growth charts (9, 10) which offered percentiles for all measurements in all ages and allowed a longitudinal confrontation.

Eating habits are intertwined with neurodevelopment evolution and weaning is a pediatric strategic knot. Our DS children did not show major delays at weaning start. Delays occurred only in those who underwent surgical interventions, especially for congenital heart diseases, during their 1st months of life. Even after a good start, however, the weaning progress was not easy. Sippy cups and glasses were introduced between 12 and 24 months of age in all children but one. However, only one child every five was able to independently drink from the cup or glass by 24 months of age. This threshold should be achieved based on the International Pediatric Association's recommendations to prevent health risks such as overweight (19). Chewing also appeared to be critical; parents struggled in the selection of food consistencies for several years after weaning started. Overall, these results trace what described in previous studies (4). Intervention programs were suggested, but not much has been published aside from Pelchat's study in 2010 (20). At that time, she demonstrated the positive achievements of families attending a counseling program with high levels of competencies and attitudes. Our study seems to confirm Pelchat's study by showing that the families who had entered the support program at an early age of their children and were followed for a long time experienced fewer problems. It also proves that larger studies and confrontations among programs are needed.

Parents seem to believe that DS children need nutrition supplements because they eat food poor in fibers, vitamins, micronutrients, and antioxidants to bypass the chewing and swallowing difficulties. In a recent survey, almost 50% of interviewed parents gave supplements to their DS children (more than 80% under 2 years of age), and one in five did not inform the pediatrician (21). This pattern was absent in our cohort, even for zinc, which was the only laboratory deficient value. Considering that, compared to recommendations, we found an appropriate and sometimes even excessive oral introduction, nutrition supplements seem unnecessary. Adequate zinc intakes were also found in a group of Brazilian DS children (5). Given the importance of zinc, especially in immune function regulation at the skin and gastrointestinal levels (these two conditions alone covered over 60% of the medical problems in this group), intracellular and urinary zinc levels should be further investigated.

Looking more specifically at nutrients, eating habits were not despicable either. Our cohort had similar eating patterns as other DS children compared to the general pediatric population (4, 5, 16). They had high protein and saturated fat intakes, low calcium and fiber intakes, and no excessive overall caloric intake. The gap between recommended and actual intakes of calcium increased with the children's age, exposing the adolescents to a potential impaired peak bone mass. The same trend was found for fiber, sodium, and folate. Diet patterns were more appropriate in preschool children, as the role played by parents is stronger. Parents also appeared not to be afraid of offering foods high in fiber to their toddlers, despite chewing difficulties. Luke et al. compared diets of DS children and controls without DS, finding that both groups assumed excessive amounts of proteins and insufficient amounts of fiber compared to recommendations (16). Calcium intake was not considered. Energy expenditure was measured and compared to the reported and the recommended intakes, both in the DS and the control groups. In doing so, they proved that DS children burned less calories than controls and their recommendations were too high. It is possible that this lower energy expenditure may be ascribed to lower VO2max (16). A reduced if not absent puberty peak could be another reason. As a general suggestion, energy recommendations should be reconsidered.

Fruit juices and ready to drink tea were the main sources of simple sugars. Their intakes increased with age and especially in boys, although soft drinks were rarely assumed. It is possible that the observed significantly increasing trend of triglycerides in school age children may depend on fructose intake, largely present in fruit juices and teas. The effects of fructose on liver steatosis and hypertension occurrences have already been confirmed in the literature (22). Liver ultrasound however was not available in our group.

We are aware that this study has some limitations. First, the group is small and the age range is wide. However, DS is still a rather rare condition and the literature presents studies with similar sample sizes. In addition, the information we collected is less heterogeneous than the age-range would imply, because the parents of these children were all very committed and it was they who provided most of the data. Therefore, the differences we found in the three age subgroups cannot be ascribed to the children's different reporting abilities, and they merit further exploration. Unfortunately, we could not carry out further statistical analysis in the three subgroups due to their small numbers. Second, nutrient intakes were self-reported through food diaries. To limit potential biases, the food diaries were verified through direct interviews with the dietician who had followed the children during the entire time span of the study, offering information and education tools to the families. The dietician also verified and cross-checked the information, combining the 3-day food frequency questionnaire and the 24 h recall. We had the impression that the parents of these children had a good nutrition knowledge, which may have contained biases during data collection. As for the program, there is room for improvements. Meals, for instance, were investigated at the personal level. The next step can be sharing food with other children, instructors, and families. Third, some nutrients such as vitamins D and C were not evaluated, mostly due to the lack of laboratory data. Fourth, the children in this sample reached a level of nutritional, social, and psychomotorial development closer to that of the general pediatric population. Approaches were similar across all patients, offering objective and comparable data and selection bias minimization, both of which enhanced the quality and credibility of the results. However, it is necessary to verify and quantify the potential benefits in the general DS population as well as to confront the benefits of DS childhood participation in different organized support programs.

This study offers several practical suggestions. Anthropometrically, the risk of overweight/obesity can be curtailed in DS. Nutritionally, eating habits can reach good standards and future support programs may tailor nutrition patterns with a special attention to saturated fats, simple sugars, fiber, micronutrients and vitamins intakes. Developmentally, the early and late phases of the weaning process are critical and need to be monitored step by step especially in children affected by prolonged hospital stays and surgical operations. Fifty-three percent of the families reported no nutrition counseling when entering the program and 47% relied on different sources including Internet. This may have delayed the weaning process. Therefore, interventions are mandatory and may include sections in which children share meals. This aspect may also prove psychologically useful to parents. Their influence on child eating behaviors has been widely recognized (23). The reverse may also occur as children with disabilities may influence what the family eats, the structure of meals at home, and the parental stress during meals (24).

## Conclusions

In conclusion, aspects in lifestyles of DS children attending a small care program seem different than what present in the literature. Nutritionally and developmentally, these children, especially those in early ages, seem to achieve better results. Only few corrections emerged as necessary, such as (i). to contain protein and saturated fat intakes at all ages, (ii). to adjust sodium, calcium, folate, and fiber in children entering adolescence, and (iii). to suggest and start prompt intervention in the early years of DS children. Not only hospital-based approach, but practical yet professional suggestions through a small reality such as this care program may accompany DS children and their families. This small reality may be exported and adapted worldwide.

This could make a difference in terms of both treatments and prevention of “future” events. We do hope that this exploratory study may act as a stimulus for larger, multicenter endeavor.

## Data Availability Statement

The raw data supporting the conclusions of this article will be made available by the authors, without undue reservation.

## Ethics Statement

The studies involving human participants were reviewed and approved by Insieme no profit association. Written informed consent to participate in this study was provided by the participants' legal guardian/next of kin.

## Author Contributions

GR and MF: conceived, coordinated, and carried out the study including statistical analyses and manuscript drafting. GC, RD, AC, GB, and MS: collaborated in the evaluation. All authors participated in the manuscript and took public responsibility for its content, contributed to drafting, and revising the paper.

## Conflict of Interest

The authors declare that the research was conducted in the absence of any commercial or financial relationships that could be construed as a potential conflict of interest.

## Publisher's Note

All claims expressed in this article are solely those of the authors and do not necessarily represent those of their affiliated organizations, or those of the publisher, the editors and the reviewers. Any product that may be evaluated in this article, or claim that may be made by its manufacturer, is not guaranteed or endorsed by the publisher.

## Abbreviations

DS: down syndrome
TC: total cholesterol
HDL: high-density lipoprotein cholesterol
LDL: low-density lipoprotein cholesterol
TG: triglycerides
LARN: recommended nutrient intakes for the Italian population
BMI: body mass index
